# Genetic Association between TNFA Polymorphisms (rs1799964 and rs361525) and Susceptibility to Cancer in Systemic Sclerosis

**DOI:** 10.3390/life12050698

**Published:** 2022-05-07

**Authors:** Joanna Kosałka-Węgiel, Sabina Lichołai, Sylwia Dziedzina, Mamert Milewski, Piotr Kuszmiersz, Anna Rams, Jolanta Gąsior, Aleksandra Matyja-Bednarczyk, Helena Kwiatkowska, Mariusz Korkosz, Andżelika Siwiec, Paweł Koźlik, Agnieszka Padjas, Wojciech Sydor, Jerzy Dropiński, Marek Sanak, Jacek Musiał, Stanisława Bazan-Socha

**Affiliations:** 1 Department of Internal Medicine, Jagiellonian University Medical College, 30-688 Kraków, Poland; sabina.licholai@uj.edu.pl (S.L.); sylwiaz49@poczta.fm (S.D.); mamert.mmm@gmail.com (M.M.); piotrkusz@gmail.com (P.K.); a.m.w.rams@gmail.com (A.R.); jgasiorr@gmail.com (J.G.); aleksandra.matyja-bednarczyk@uj.edu.pl (A.M.-B.); lek.andzelika.siwiec@gmail.com (A.S.); pawelkozlik89@gmail.com (P.K.); agnieszka.padjas@uj.edu.pl (A.P.); wjsydor@gmail.com (W.S.); jerzy.dropinski@uj.edu.pl (J.D.); marek.sanak@uj.edu.pl (M.S.); jacek.musial@uj.edu.pl (J.M.); stanislawa.bazan-socha@uj.edu.pl (S.B.-S.); 2 Rheumatology and Immunology Clinical Department, University Hospital, 30-688 Kraków, Poland; mariusz.korkosz@uj.edu.pl; 3 Department of Rheumatology and Immunology, Jagiellonian University Medical College, 30-688 Kraków, Poland; 4 Outpatient Clinic for the Immunological and Hypercoagulable Diseases, University Hospital, 30-688 Kraków, Poland; 5 Department of Dermatology, University Hospital, 30-688 Kraków, Poland; hkwiatkowska7@gmail.com; 6 Department of Hematology, University Hospital, 30-688 Kraków, Poland

**Keywords:** TNFA, systemic sclerosis, Scl, Scl-ILD, genetic association

## Abstract

Tumor necrosis factor (TNF)-α is a proinflammatory cytokine that plays an important role in the pathogenesis of autoimmune diseases. The aim of the study was to establish an association between TNF-α promoter variability and systemic sclerosis (SSc). The study included 43 SSc patients and 74 controls. Four single nucleotide polymorphisms (rs361525, rs1800629, rs1799724, and rs1799964) located at the promoter of the TNFA gene were genotyped using commercially available TaqMan allelic discrimination assays with real-time PCR. The rs1799724 allele was associated with an increased SSc susceptibility (*p* = 0.028). In turn, none of the polymorphisms studied were related to the clinical and laboratory parameters of SSc patients, except for a higher prevalence of anti-Ro52 antibodies in the AG rs1800629 genotype in comparison to GG carriers (*p* = 0.04). Three of four cancer patients had both CT rs1799964 and AG rs361525 genotypes; thus, both of them were related to the increased risk of cancer, as compared to the TT (*p* = 0.03) and GG carriers (*p* = 0.0003), respectively. The TNFA C rs1799724 variant is associated with an increased risk of SSc, while the CT rs1799964 and AG rs361525 genotypes might enhance cancer susceptibility in SSc patients, although large observational and experimental studies are needed to verify the above hypothesis.

## 1. Introduction

Systemic sclerosis (SSc, scleroderma) is a complex multiorgan disease characterized by vascular damage, perivascular inflammation, the presence of specific autoantibodies and the progressive fibrosis of the skin and internal organs [1,2,3]. The initial vascular damage seems to precede and provoke organ inflammation, followed by the accumulation of fibrotic collagen and other extracellular matrix components in vessel walls and interstitial tissue [4]. Among the cytokines involved in SSc pathogenesis, tumour necrosis factor (TNF)-α has been implicated as playing a pivotal role in the inflammatory process, in part through up-regulating mTOR gene expression by the activation of the NF-κB pathway [4,5,6,7]. TNF-α, which is produced mainly by macrophages, disrupts vascular endothelial cell–cell junctions and promotes monocyte adhesion to the endothelial cells [5,8]. SSc shows substantial heterogeneity in its clinical symptoms, patterns of organ involvement, and natural history [9]. It may have limited involvement (lcSSc) in calcinosis cutis, Raynaud’s phenomenon, oesophageal dysmotility, sclerodactyly, and telangiectasia (also known as CREST syndrome); or diffuse involvement (dcSSc) in the higher risk of early lung fibrosis and acute renal involvement [3,10]. CREST syndrome features may also develop in dsSSc, which is usually associated with a worse overall prognosis [10]. Moreover, serological differences have been detected [11]. In this regard, anticentromere antibodies (ACA) are more specific for lcSSc while anti-DNA topoisomerase I (Scl-70) antibodies show an increased prevalence in dcSSc (but are also detected in lcSSc) [12]. Although the aetiology of SSc is largely unknown, epidemiological investigations indicate that SSc follows a pattern of multifactorial inheritance, with various risk and protective loci contributing to disease predisposition and clinical phenotype together with epigenetic and environmental factors [2,11,13,14]. Recent candidate gene and genome-wide association studies have identified several genetic variants that are associated with SSc susceptibility, including those of the TNF-α promoter [2].

Taking into account an important role of TNF-α in the pathogenesis of inflammatory response and SSc progression [3,15,16], and the fact that the *TNFA* gene promoter has several single nucleotide polymorphisms (SNPs) [5], we decided to analyse whether those genetic variabilities might contribute to SSc susceptibility, including the phenotypic expression of the disease [12]. To address this topic, we studied four potentially functional SNP polymorphisms (rs361525, rs1800629, rs1799964, and rs1799724) located within the *TNFA* gene promoter among the SSc cohort and compared them to a control group.

## 2. Materials and Methods

### 2.1. Participants

The studied group consisted of 43 SSc patients, 31 women and 12 men, with a median age of 59, who were recruited in the period from 2014 to 2020 at the Department of Allergy and Clinical Immunology and Department of Dermatology, University Hospital, Cracow, Poland. All patients fulfilled the 2013 European League Against Rheumatism/American College of Rheumatology (EULAR/ACR) classification criteria or the criteria for the diagnosis of Early SSc (EaSSc) proposed by LeRoy and Medsger [17,18]. Subjects with comorbidities, including arterial hypertension, diabetes mellitus or hypercholesterolemia were eligible for participation in the study. Hypertension was defined as a history of blood pressure ≥ 140/90 mmHg or the preadmission of antihypertensive treatment [19,20]. Diabetes mellitus was defined as a condition treated with insulin or oral hypoglycaemic agents or having a fasting serum glucose ≥ 7.0 mmol/L [19,21]. Hypercholesterolemia was defined as previously diagnosed and treated with statins, having a serum total cholesterol ≥ 5.0 mmol/L, or having a serum low-density lipoprotein ≥ 3.0 mmol/L [19,22]. All the study subjects were analysed for anticentromere (ACA), anti-topoisomerase I (Scl-70) and antinuclear (ANA) antibodies, using indirect immunofluorescence staining (IIF) and/or the immunoblot technique. The presence of SSc-associated interstitial lung disease (SSc-ILD) was diagnosed based on typical findings on chest high-resolution computed tomography (HRCT). The presence of pulmonary arterial hypertension (PAH) was defined as pulmonary artery systolic pressure > 45 mmHg, which was shown to have 95% specificity vs. right heart catheterization, which is considered a gold standard for the diagnosis of PAH [11]. Scleroderma renal crisis (SRC) was defined as a new renal insufficiency with or without arterial hypertension that could not be explained by reasons other than SSc. Digital ulcers (DUs) were defined as painful areas with a loss of tissue distal to the proximal interphalangeal joint (PIP) digital crease. The 38-individual control group was made up of 28 women and 10 men that matched the case group in terms of gender (Table 1).

The study received approval from the Bioethics Committee of Jagiellonian University Medical College. All subjects were given a thorough description of the methodology and safety protocol before we obtained their written consent to participate in the study.

### 2.2. Genotyping

Blood samples were collected from all subjects of the study. The samples were kept at 4 °C until DNA extraction, which was performed within 6 h of blood collection. DNA was extracted from peripheral leucocytes using DNAzol reagent (Invitrogen Life Technologies, Carlsbad, CA, USA). Four SNPs located in the promotor of *TNFA* gene were selected, namely rs361525, rs1800629, rs1799964, and rs1799724. SNPs were genotyped with commercially available TaqMan allelic discrimination assays (Life Technologies) using the 7900 HT Real-time PCR System (Applied Biosystems, Foster City, CA, USA). A mix of unlabelled PCR primers and TaqMan MGB probes labelled with FAM or VIC dye were used. The reaction was performed in a 10-μL solution that contained 0.5 μL of a 40× oligonucleotides mix, 5 μL of a 2 × TaqMan Genotyping Master mix (Applied Biosystems, all), and 2 μL of 50 ng genomic DNA. PCR conditions were as follows: initial denaturation at 95 °C for 15 min; 40 cycles at 95 °C for 10 s and 60 °C for 45 s. Genotyping was performed on coded and blinded samples in the presence of negative ones (no DNA) included in each 96-well plate for quality control. The genotyping results were determined by using SDS 2.3 Allelic Discrimination Software (Applied Biosystems).

### 2.3. Statistical Analysis

A statistical analysis was performed using Statsoft Statistica v. 12 software (StatSoft, Tulsa, OK, USA) and GraphPad Prism v. 8.0 (GraphPad Software, Inc., San Diego, CA, USA). Comparisons of the SNPs frequency in the TNF-α gene in different manifestations of SSc groups were calculated according to Pearson’s goodness-of-fit test χ2. In the case of multiple testing with statistically significant results, Bonferroni correction was applied. Fisher’s exact test was employed when the expected frequency was less than five. Data distribution was evaluated using the Shapiro–Wilk test. All continuous variables were non-normally distributed, and were thus presented in the manuscript as a median with an upper and lower quartile, as well as with a 95% confidence interval of the standard deviation, and compared with a Mann–Whitney U-test, Kruskal–Wallis test or multiple repetition test as appropriate. A one-way covariance analysis (ANCOVA) was performed to adjust for potential confounders, including age, sex and body mass index (BMI). A *p*-value < 0.05 was considered significant.

## 3. Results

### 3.1. Patients’ Characteristics 

As is presented in Table 1, both groups of subjects were quite similar in demographic variables, except age. 

Table 2 shows the clinical parameters and the presence of autoantibodies in SSc patients. As is presented, the median duration of the disease was 4 (range: 1–9) years and the majority of patients (*n* = 26, 60.5%) were diagnosed with a diffuse type of the disease. Raynaud’s phenomenon was the most frequent clinical sign reported in 95.3% of the cases. ILD was found in nearly 70% of SSc patients, while other manifestations, including dysphagia, PAH and digital ulcers, were less frequent. More than one third of patients were currently treated with steroids, while 26 (60.5%) subjects were either receiving or had previously received immunosuppressive regimens, such as methotrexate, azathioprine, cyclophosphamide, and mycophenolate mofetil. Patients also received other medications, such as calcium channel blockers, angiotensin converting enzyme inhibitors or angiotensin receptor antagonists, as well as statins (Table 2).

Antinuclear antibodies were detected in all patients and anti-DNA topoisomerase I was detected in 26 (60.5%), whereas ACA and anti-Ro52 were detected in nearly a quarter of patients. Other types of antibodies, such as anti-RNA polymerase III, anti-PM/Scl anti-NOR, anti-Th/To and anti-Ku were detected at a much lower frequency.

It is worth noting that in the SSc group four patients were diagnosed with cancer. Two of them had lung cancer, one subject was diagnosed with carcinoma planoepitheliale confirmed in a histological examination of lymph nodes (cancer of unknow origin), and one patient had breast cancer and multiple myeloma.

### 3.2. Distribution of TNFA Polymorphisms in SSc Patients and Controls

The allele frequencies of the *TNFA* promoter for rs1799724, rs1800629, rs1799964, and rs361525 polymorphisms in the SSc patients and controls are shown in Table 3. The T rs1799724 genotype variant was associated with an increased SSc susceptibility (*p* = 0.028). An analogous tendency was not observed by us between rs1800629, rs36152 and rs1799964 polymorphisms and SSc.

### 3.3. TNFA Polymorphisms and Clinical Parameters in SSc 

We analysed the relationship between all studied *TNFA* promoter polymorphisms and the clinical and laboratory parameters of SSc patients. However, none of them were associated with the clinical and laboratory parameters of the disease, except for a higher prevalence of anti-Ro52 antibodies in the AG rs1800629 genotype in comparison to GG carriers (*p* = 0.0398); the AA genotype was not detected in the SSc group. 

### 3.4. TNFA Polymorphisms and Comorbidities in SSc 

The rs1799724, rs1800629, rs1799964 and rs361525 polymorphisms had no significant associations with hypertension, diabetes mellitus, and hypercholesterolemia in SSc patients. However, three of four cancer patients had both CT rs1799964 and AG rs361525 genotypes, thus, both of them were found to be related to the increased risk of cancer, as compared to the TT (*p* = 0.03) and GG carriers (*p* = 0.0003), respectively (Table 4 and Table 5). The cancer group did not differ in disease-related factors and comorbidities in comparison to the remaining SSc individuals.

## 4. Discussion

In the present study, we demonstrated that in the Polish population there exists an association between particular *TNFA* genotypes and the incidence of SSc, and, furthermore, specific clinical manifestations can be associated with specific alleles and their arrangements. 

The potential strength of this association is reinforced by the possible biological involvement of this cytokine in the pathogenesis of the disease. TNF-α participates in the activation of the vascular endothelium, regulation of the immune response and is implicated in type I collagen production by fibroblasts, with antifibrotic activity, and thus may be important in SSc pathogenesis [3,12,23]. The interest in SSc has also been motivated by the possible role of TNF-α in potentiating the PAF (platelet activating factor)-induced vasoconstriction in the pulmonary circulation, since ischemia and reperfusion are major determinants of lung endothelial injury [24,25,26]. Moreover, TNF-α increases the gene expression of low-density lipoprotein receptor 1 (LOX-1); this receptor can support the adhesion of bacteria to vascular endothelial cells and macrophage cells, thereby suggesting its role in inflammation and vascular injury [27,28,29]. TNF-α also induces the gene expression of vascular cell adhesion molecule 1 (VCAM-1) which play a crucial role in rolling-type mononuclear cell adhesion and neo-intima formation in case of injury and smooth muscle cell proliferation [30,31,32]. Furthermore, many other endothelial surface cell adhesion molecules, including endothelial-leucocyte adhesion molecule-1 (ELAM-1), E-selectin, P-selectin and intracellular adhesion molecule 1 (ICAM-1), are increased by TNF-α, facilitating adhesion, the ingress of eosinophils and T cells to the inflammatory locus and an increase in microvascular permeability [7,32,33,34,35].

In view of the evidence, TNF-α might be a major contributor to SSc progression due the overall vascular damage [3,12]. Some studies on TNFA polymorphisms have reported an association between the rs361525 and rs1800629 polymorphisms with SSc risk in different populations, nevertheless, its role in the Polish population was previously unknown [3,12,36]. Moreover, there are some data describing the association between some SNPs (e.g., TNFα promoter T rs1799724 genotype variant is higher than C rs1799724 genotype variant in the levels of transcriptional activity of the gene, mRNA, and protein of the TNFα) and mRNA or protein production [37]. In the present study, we investigated whether single nucleotide polymorphisms (SNPs) in the promoter of the TNF-α gene have an association with SSc and its clinical and serological subsets. We found that the T rs1799724 genotype variant was associated with an increased SSc susceptibility (*p* =0.028). A similar strong association was described previously in the case of other autoimmune diseases, including autoimmune hepatitis, Behcet’s or Crohn’s disease, and also cancer [38,39,40,41]. However, we did not observe an analogous tendency between rs1800629 and rs1799964 polymorphisms and SSc. These results are different to those reported in other populations [3,15,36]. Lomeli-Nieto JA et al. highlighted an association of the AG rs1800629 genotype with disease susceptibility according to not only a codominant genetic model, but also with higher anti-fibrillarin antibodies, and higher skin thickening [3]. In some studies, authors underlined that the AG rs1800629 genotype is associated with higher TNFA mRNA expression than GG genotype carriers, which could subsequently skew the immune response toward a more deleterious outcome [3,42,43,44,45]. On the other hand, the genetic research investigating the link between TNFA rs1799964 polymorphisms and autoimmune diseases is very limited and mainly concerns patients suffering from rheumatoid arthritis (RA), colitis ulcerosa (CU) or Crohn’s disease [46,47]. Interestingly, we observed that SSc patients with the CT rs1799964 genotype in comparison to the homozygous T carrier were characterized with a higher frequency of cancers. Additionally, Oh SS et al. underlined the association of rs1799964 SNPs with smoking-related cancers among never smokers, whereas Li X et al. highlighted the correlation with cervical cancers [48,49]. We did not find an association between rs361525 polymorphisms and disease risk, which is in line with data presented by Lomeli-Nieto JA et al. and Ates O et al. [3,36]. Interestingly, Lomelli-Nieto JA et al. observed that SSc patients carrying the AG rs361525 genotype have been associated with higher serum TNF-α (sTNF-α) levels and had ~4.5-fold higher TNFA mRNA expression than GG genotype carriers [3]. In contrast to our findings, a study performed in Italy reported an association between the AG rs361525 genotype and SSc susceptibility, particularly in patients with the dSSc phenotype [12]. Moreover, Tolusso B et al. did not confirm an association between the serological subsets (presence of ACA or anti-Scl-70 autoantibodies) and the considered polymorphisms in an Italian population [12]. These heterogeneous associations between TNFA polymorphisms and SSc risk can be explained by the population size and genetic structure of each evaluated population, which, as is well known, contributes to differences in disease incidence between populations. All of the patients with the AG rs361525 genotype were characterized with the presence of cancer. Half of the SSc patients with cancers were diagnosed with lung cancer (both presented with AG rs361525 genotype). In contrast to our results, the data presented by Qidwai T et al. suggested that individuals with AA/GA rs361525 genotypes compared to those with the GG genotype had a lower odds ratio for lung cancer; moreover, a GG genotype had a tendency to advance disease progression [50]. Additionally, data presented by Liu N et al. indicate that rs361525 GG genotype carriers contribute to a higher risk of lung squamous cell carcinoma [51]. Zmorzyński Sz et al. observed that the presence of rs361525 AA or AG genotypes had an increased risk of multiple myeloma (MM) and is associated with an earlier onset of the disease. Contrary to those results, SSc patient diagnosed with MM were characterized by the GG genotype [52]. Published data about the association of TNFA rs361525 polymorphisms with breast cancer still remain inconsistent [50,53,54]. The relationship between cancers risk and TNFA rs361525 polymorphisms has been demonstrated but remains controversial [50,51,52,53,54,55,56,57].

Of note, our study has several limitations. Firstly, the analyzed group of SSc patients is small. Particularly, subgroup analyses need to be performed with caution. However, our study, which included 43 SSc patients from one center is unique and, in our opinion, valuable in the context of *TNFA* SNPs and SSc susceptibility. Additionally, we did not analyzed TNFa or other cytokines in serum. 

## 5. Conclusions

Based on our findings, we suggest that *TNFA* rs1799964 and rs361525 variant genotypes are associated with cancer susceptibility in SSc. However, our results should be verified in the future in a larger group of SSc patients.

## Figures and Tables

**Table 1:** A summary of demographic and clinical characteristics of subjects studied.

	Patients *n* = 43	Controls *n* = 38	*p*-Value
Age, years	59 (53.3–61)	46.50 (41–50)	0.008
Male gender, number (%)	12 (27.9)	10 (26.31%)	ns
Body mass index, kg/m^2^	24 (23.6–26.1)	n.a.	n.a.
Smoking in the past, number (%)	16 (37.2)	n.a.	n.a.
Smoking, packs/years	0 (0–0.5)	n.a.	n.a.

Categorical variables are presented as numbers (percentages), continuous variables as median and 95% confidence interval of standard deviation. Abbreviation: n.a.—not analyzed.

**Table 2:** Clinical characteristics and antinuclear antibodies in systemic sclerosis patients.

	Patients *n* = 43
Duration of the disease (years)	4 (1–9)
Limited disease, n(%)	17 (39.5)
Diffuse disease, n(%)	26 (60.5)
The presence of anti-nuclear antibodies, n(%)	43 (100)
Anti-Scl-70 antibodies presence, n(%)	26 (60.5)
Anti-PM/Scl antibodies presence, n(%)	6 (14)
Anti-centromeric antibodies presence, n(%)	10 (23.5)
Anti-NOR antibodies presence, n(%)	3 (7)
Anti-Ro52 antibodies presence, n(%)	9 (20.9)
RNA polymerase III antibodies presence, n(%)	1 (2.3)
Anti-Th/To antibodies presence, n(%)	1 (2.3)
Anti-Ku antibodies presence, n(%)	1 (2.3)
**Organ involvement**	
Digital ulcers, n(%)	14 (32.6)
Abnormal nailfold capillaries, n(%)	25 (58.1)
Telangiectasia, n(%)	11 (25.6)
Raynaud’s phenomenon, n(%)	41 (95.3)
Dysphagia, n(%)	11 (25.6)
Interstitial lung disease, n(%)	30 (69.8)
Pulmonary arterial hypertension, n(%)	10 (23.3)
**Treatment characteristic**	
Current steroids therapy, n(%)	17 (39.5)
Current corticosteroid dose, mg per day, recalculated to methylprednisolone	0 (0–4)
Systemic steroids therapy, years	0 (0–3.3)
**Immunosuppressive treatment (currently or in the past)**	
Azathioprine, n(%)	5 (11.6)
Cyclophosphamide, n(%)	15 (34.9)
Methotrexate, n(%)	12 (27.9)
Mycophenolan mofetil, n(%)	5 (11.6)
**Comorbidities**	
Hypertension, n(%)	20 (46.5)
Diabetes mellitus, n(%)	3 (7)
Hypercholesterolemia, n(%)	19 (44.2)
**Internal medicine medications**	
Angiotensine converting enzyme inhibitors or angiotensin receptor antagonists, n(%)	21 (48.8)
Statins, n(%)	14 (32.6)
Beta-blockers, n(%)	11 (25.6)
Diuretics, n(%)	12 (27.9)
Calcium channel blockers, n(%)	26 (60.5)

Categorical variables are presented as numbers (percentages), continuous variables as median and interquartile range (25–75Q). Abbreviation: n—number.

**Table 3:** Frequencies of particular genotypes and alleles in the SSc group and controls.

SNP	Genotype	Patients *n* = 43	Controls *n* = 38	*p*-Value
rs1799724	CC	27 (62.8)	34 (89.4)	
	CT	13 (30.2)	4 (0.10)	0.028 *
	TT	3 (7)	0	
rs1800629	AA	0 (0)	1 (2.63)	
	AG	14 (32.6)	6 (15.78)	0.137
	GG	29 (67.4)	31 (81.57)	
rs1799964	CC	0 (0)	1 (2.63)	
	CT	10 (23.3)	8 (21.05)	0.55
	TT	33 (76.7)	29 (76.31)	
rs361525	AA	0 (0)	0	
	AG	3 (7)	2 (5.26)	0.95
	GG	40 (93)	36 (94.73)	

Categorical variables are presented as numbers (percentages). Statistically significant results are marked *.

**Table 4:** Frequencies of cancers in *TNFA* genotypes in the SSc group.

SNP	Genotype (Number of SSc Patients)	Patients with Cancer *n* = 4	*p*-Value
rs1799724	CC (27)	14.8 (4)	*p* = 0.2707
	CT (13)	0 (0)	
	TT (3)	0 (0)	
rs1800629	AG (14)	21.4 (3)	*p* = 0.0936
	GG (29)	34.5 (1)	
rs1799964	CT (10)	30 (3)	*p* = 0.0338 *
	TT (33)	3 (1)	
rs361525	AG (3)	100 (3)	*p* = 0.0003 *
	GG (40)	2.5 (1)	

Categorical variables are presented as percentages (numbers). Statistically significant results are marked *.

**Table 5:** Characteristics of systemic sclerosis patients with diagnosed cancer.

	Cancer	Time from SSc Diagnosis to Cancer Diagnosis (Years)	rs1799964 Genotype	rs361525 Genotype
Patient 1	lung cancer	19	CT	AG
Patient 2	lung cancer	15	CT	AG
Patient 3	cancer of unknow origin	4	CT	AG
Patient 4	breast cancer *	0	TT	GG

* Patient no 4 was also diagnosed with multiple myeloma (1 year after SSc diagnosis).

## Data Availability

The datasets analyzed are available from the corresponding author upon reasonable request.
